# Senescence‐Associated *Cdkn1a* (p21) Is Upregulated in Rodent Dorsal Root Ganglion Neurons Across Lesion‐Based Neuropathic Pain Models

**DOI:** 10.1155/prm/6836731

**Published:** 2026-10-25

**Authors:** Philipp N. Ostermann, Rebecca Dinnendahl, Jörg Isensee, Tim Hucho

**Affiliations:** ^1^ Translational Pain Research Department of Anaesthesiology and Intensive Care Medicine Faculty of Medicine and University Hospital Cologne University of Cologne 50931 Cologne Germany uni-koeln.de

**Keywords:** Cdkn1a, Cdkn2a, chronic pain, Dorsal root ganglion, neuropathic pain, p16, p21, peripheral sensory neuron, senescence

## Abstract

**Background:**

Senescence of peripheral sensory neurons has recently been associated with increased nociceptive signalling and pain chronification. Whether increased expression of senescence‐associated genes is found across pain conditions and independent study settings remains to be investigated.

**Methods:**

We analysed 20 publicly available whole‐genome transcriptome data sets on rodent dorsal root ganglion (DRG) tissue and focused on *Cdkn1a* (p21) and *Cdkn2a* (p16) expression levels as two widely used senescence markers in different pain models.

**Results:**

We found that *Cdkn1a* but not *Cdkn2a* RNA expression exhibited a robust recurrent significant increase in different axotomy‐ and lesion‐based neuropathic pain models but not in other pain models, including inflammatory pain. Data from one study including male and female rats indicated a potential sex‐dependent effect of *Cdkn1a* upregulation following nerve injury, which warrants further investigation. Overall, *Cdkn1a* RNA levels were increased among all DRG neuronal subtypes, seemed to have reached their maximum three to 7 days following nerve injury and remained elevated in some neurons as long as 60 days post‐injury.

**Conclusions:**

*Cdkn1a* upregulation in DRG neurons is a widespread response to nerve injury that is found across independent study settings.

**Significance Statement**
• By integrating multiple independent transcriptomic data sets, we show that *Cdkn1a*, commonly used as a senescence‐associated marker, is consistently elevated in dorsal root ganglia after nerve injury, particularly in neurons. While not sufficient to define neuronal senescence, these findings indicate that injury‐related transcriptional changes linked to senescence pathways may be more prominent in neuropathic than in other pain models, informing future mechanistic and therapeutic investigations.

## 1. Introduction

Classical cellular senescence is an apoptosis‐resistant state of irreversible cell cycle arrest in mitotic cells, induced by various stressors to prevent proliferation of damaged cells [1, 2]. It is involved in tissue repair and preventing cancer, but also associated with the development of a senescence‐associated secretory phenotype (SASP), which is characterised by the secretion of proinflammatory, proapoptotic and profibrotic factors that can disturb tissue function and turn nearby cells themselves senescent [1].

Postmitotic nonproliferating neurons have been found to adopt a senescence‐like state (previously proposed as ‘neurescence’ [3]) in response to stressful environments [2, 4]. Senescent neurons are metabolically active, but show altered excitability and transition into the potentially contagious state of SASP similar to non‐neuronal cells [2, 3]. Senescence of central nervous system neurons is increasingly associated with multiple neurodegenerative disease such as Alzheimer’s and Parkinson’s [5, 6].

Neuronal senescence through ageing and injury has recently been suggested as a mechanism, leading to increased activity in peripheral sensory neurons and to pain chronification [7–9]. Reducing the rates of senescent peripheral sensory neurons with senolytics has successfully alleviated nocifensive behaviour in two rodent neuropathic pain models so far [8, 9]. Corroborating cellular senescence as a core aspect of lasting pain and identifying cellular markers thereof could open the way to a new class of analgesic drugs soon.

Cyclin‐dependent kinase inhibitors CDKN1A (p21) and CDKN2A (p16)—and their RNA expression levels—have been reported as markers for cellular senescence, both of which play a role in cell state stability, and in induction and maintenance of cellular senescence [2, 10]. Notably, CDKN1A (p21) is also involved in cellular stress pathways, DNA damage responses and regeneration. Expression induced in cancer cells by chemotherapy was found to have the capacity to induce either senescence or proliferation [11]. An early increase in p21 did not automatically lead to cellular senescence, supporting the notion that additional assays are required to define senescence [11]. However, among all senescence markers summarised by the SenNet recommendations, CDKN1A and CDKN2A exhibited the broadest tissue distribution. Specifically, their expression was associated with senescence across 15 and 14 distinct tissues, respectively, including the lungs, liver, kidneys, skin, skeleton, pancreas, bone marrow and, importantly, the central nervous system [10]. Given that CDKN1A and CDKN2A expression is poorly defined for peripheral sensory neurons, but they harbour the potential role of inducing senescence, it remains an important task to map their expression patterns in the context of different pain models. Moreover, the adoption of neuronal senescence is a complex, time‐dependent process and senescence markers may be differentially regulated in different neuronal subtypes, which can be studied with available scRNA sequencing data [3].

Proof of principle for a role of peripheral sensory neuron senescence for pain has been provided [8, 9]. The extent to which peripheral sensory neurons express senescence markers across different pain conditions, and different sensory neuron subtypes remains unclear. Therefore, we made use of publicly available whole‐genome microarray, bulk‐ and single‐cell (sc)RNA sequencing data sets from previous studies on rodent dorsal root ganglion (DRG) tissue to investigate pain‐related upregulation of senescence markers *Cdkn1a* and *Cdkn2a* in peripheral sensory neurons across different, independent study settings. Such an approach is of high relevance due to the larger number of samples, the diversity of pain models and, moreover, the independence of the performed studies.

## 2. Methods

### 2.1. Sex and Gender

Sex was considered a biological factor. Publicly available studies were analysed that used male, female or male and female animals. We specifically addressed and discussed a potential role of sex in our study based on the available data sets.

### 2.2. Systematic Data Set Selection Methodology

To identify relevant gene expression data sets, a systematic literature search was conducted (Supporting Figure 1). The digital repository PubMed was searched for articles including relevant data sets up to January 2026. The search strategy employed combinations of terms and text words related to the tissue (e.g., ‘Dorsal root ganglion’, ‘DRG’, ‘Sensory neuron’, ‘Somatosensory neuron’, ‘Peripheral neuron’, ‘Nociceptor’), condition (e.g., ‘Pain’, ‘Chronic pain’, ‘Nociception’, ‘Neuropathy’, ‘Inflammatory pain’), method (e.g., ‘Sequencing’, ‘RNAseq’, ‘Bulk‐RNAseq’, ‘scRNAseq’, ‘Single cell RNA’, ‘Microarray’, ‘Transcriptomic analysis’, ‘Whole‐genome sequencing’) and organism (e.g., ‘Mouse’, ‘Rat’, ‘Rodent’). In addition, the reference lists of retrieved research articles and relevant review articles were manually screened to identify further eligible studies. Studies were eligible for inclusion if they met the following criteria: (1) Only newly generated data sets from original studies were included; (2) the study used mice (*Mus Musculus*) or rats (*Rattus norvegicus*); (3) the study analysed gene expression in DRG tissue or DRG‐derived single cells; (4) the study analysed gene expression either in standard pain models or newly established pain models, and provided experimental data on induced nocifensive behaviour; (5) the study used microarray, bulk‐RNA, single‐cell (sc) or single‐nucleus (sn) RNA sequencing methods to analyse whole‐genome gene expression and (6) the study used tissue or cells from control animals to analyse differential gene expression. Full texts of the remaining articles were evaluated for eligibility and data accessibility. Studies were excluded if no access to transcriptomic data was provided, even after contacting the corresponding author. Ultimately, 13 studies were included. Since studies may have utilised different pain models, these 13 studies yielded a total of 20 independent data sets.

### 2.3. Use of Publicly Available Data Sets

We followed a vote‐counting strategy to analyse publicly available transcriptomic data from studies listed in Table 1. Data from Uttam et al. were retrieved from supporting tables 2 and 4 [12], from Parisien et al. from supporting tables 1, 5 and 6 [13], from Strong et al. from supporting table 2 [14], from Hu et al. from supporting table 1 and 3 [15], from Renthal et al. from supporting table 3 [16] and from Cobos et al. from supporting table 1 and the Gene Expression Omnibus (GEO) database (GSE102937) [17], from Starobova et al. from supporting table S1 [18], from Athie et al. from supporting table 1 [19] and from Calls et al. from supporting table 2 [7]. Data from Kummer et al. were downloaded from GEO (GSE104625) [20], data from Vega‐Avelaira et al. were downloaded from GEO (GSE15041) [21], and data from Stephens et al. were provided by the authors upon request; read counts were downloaded from GEO (GSE100122) [22].

**TABLE 1:** Microarray, bulk‐RNA and single‐cell/nucleus RNA sequencing studies analysed.

Study	Year	Species	Age/genotype/sex	Tissue	Intervention	Chronic pain type	Timepoint(s)	Sequencing approach
Athie et al.	2018	*Rattus norvegicus*	7–10 weeks/Wistar/male	DRG (L4–L5)	Streptozotocin‐induced type 1 diabetes	Neuropathic pain	4 weeks	Bulk‐RNA‐seq
Berta et al.	2017	*Rattus norvegicus*	Adult/Sprague–Dawley/male	DRG (L4–L5)	Spared nerve injury (SNI)	Neuropathic pain	7 days	Microarray with LCM^a^‐dissected DRG neurons (Affymetrix GeneChip rat genome 230 v2.0 array)
Calls et al.	2021	*Mus musculus*	10 weeks/BALB/c/female	DRG	Cisplatin‐induced chemotherapy‐induced peripheral neuropathy (CIPN)	Neuropathic pain	10 weeks	scRNA‐seq
Cobos et al.	2018	*Mus musculus*	9–10 weeks/C57BL/6J/male	DRG (L4–L5)	Spared nerve injury (SNI)	Neuropathic pain	8 and 16 h, 1, 2, 3, 4, 5, 6, 7, 8, 9, 10 days	Microarray (Illumina MouseRef‐8 v2.0)
Hu et al.	2016	*Mus musculus*	2 months/CAST/Eij ♂ cross C57BL/6♀ F1 female mice and C57BL/6 of both sex	DRG (L3–L5)	Sciatic nerve transection (SNT)	Neuropathic pain	3 days	scRNA‐seq by manually picking DRG soma
Kummer et al.	2018	*Mus musculus*	20–24 weeks/C57BL/6/male	DRG (L3–L5)	*α*‐Galactosidase A deficiency as a Fabry disease model	Neuropathic pain	Aged 20–24 weeks	Microarray (Agilent SurePrint G3 Mouse GE 8 × 60K)
Parisien et al.	2019	*Mus musculus*	18 weeks/BALB/c/female	DRG (L3–L5)	Spared nerve injury (SNI) and complete Freund’s adjuvant (CFA)	Neuropathic pain, inflammatory pain	7 days (SNI), 3 days (CFA)	Bulk‐RNA‐seq
Renthal et al.	2020	*Mus musculus*	8–12 weeks/C57BL/6J/Male	DRG (lumbar)	Spinal nerve transection (SpNT), sciatic nerve crush (Crush), sciatic nerve transection + ligation (ScNT), paclitaxel (CIPN), complete Freund’s adjuvant (CFA)	Neuropathic pain, inflammatory pain	6 and 12 h, 1, 1.5, 2, 3, 7, 28, 60 days	snRNA‐seq
Starobova et al.	2020	*Mus musculus*	8–10 weeks/C57BL/6J/not provided^b^	DRG (L3–L5)	Vincristine‐ oxaliplatin‐, cisplatin‐induced chemotherapy‐induced peripheral neuropathy (CIPN)	Neuropathic pain	2 days	Bulk‐RNA‐seq
Stephens et al.	2019	*Rattus norvegicus*	Adult/Sprague–Dawley rats/both sexes analysed separately	DRG (L4–L6)	Chronic constriction injury (CCI)	Neuropathic pain	14 days	Bulk‐RNA‐seq
Strong et al.	2012	*Rattus norvegicus*	Adult/Sprague–Dawley/male	DRG (L5)	Zymosan in incomplete Freund’s adjuvant	Inflammatory pain	3 days	Microarray (Affymetrix Rat Exon ST v1.0)
Uttam et al.	2018	*Mus musculus*	8 weeks/C57BL/6J/female	DRG (L3‐L5)	Spared nerve injury (SNI)	Neuropathic pain	30 days	Bulk‐RNA‐seq
Vega‐Aveleira et al.	2009	*Rattus norvegicus*	2 months and 10 days/Sprague–Dawley/male	DRG (L4‐L5)	Spared nerve injury (SNI)	Neuropathic pain	7 days	Microarray (Affymetrix GeneChip Rat expression 230 v2.0)

^a^Abbreviation: LCM, laser capture microdissection.

^b^The study by Starobova et al. does not specify the sex of the mice used.

### 2.4. Software

Data were reanalysed using the online interactive tool GEO2R from the NCBI GEO database [23, 24] utilising the Bioconductor/R *limma* package [25], if processed data were not available as part of the original works (see below). GraphPad Prism (Version 6) and RStudio with the *ggplot2* package [26] have been used for visualisation and analysis of data. Inkscape (Version 1.2) has been used to create figures. BioRender (Licence: University of Cologne—Medical School) has been used to create schematic illustrations.

### 2.5. Statistical Analysis

Data from Parisien et al. were processed in the original work using cuffdiff. Statistical significance has been determined as *p* < 0.05, |*π*| ≥ 1.11 as previously published [27]. Data from Strong et al. were processed in the original work with default parameters of the GeneSpring software to determine statistical significance (*p* − *a* *d* *j*.<0.05) following Benjamini–Hochberg correction for multiple testing. Data from Hu et al. were processed in the original work. Statistical significance for bulk‐RNA sequencing was tested using the Wald test in DESeq2 and corrected for false discovery rates (FDRs) using the Benjamini–Hochberg [15]. Statistical significance for scRNA sequencing was determined using a Bayesian approach [28]. Data from Uttam et al. were processed in the original work following in‐house *R* scripts, as explained in Silva Amorim et al. [29] and by omitting genes with < 128 reads. Statistical significance was corrected for FDR using the Benjamini–Hochberg method [12]. Data from Stephens et al. were processed in the original work. Statistical significance was tested using the Wald test in DESeq2 and corrected for FDR using the Benjamini–Hochberg [22]. Data from Renthal et al. were processed in the original work. Statistical significance was tested using the QL F‐test in edgeR [16]. Data from Cobos et al. were processed in the original work. Statistical significance was tested using a moderated *F*‐statistic. Data from Vega‐Avelaira et al. were reanalysed using the online interactive tool GEO2R from the GEO database [23, 24]. The analysis utilised the Bioconductor/R *limma* package [25] with default settings, including log‐transformation of the data and FDR adjustment for multiple testing. Data from Starobova were processed in the original work. Statistical significance was tested using the Wald test in DESeq2 and corrected for FDR using the Benjamini–Hochberg [18]. Data from Athie et al. were processed in the original work. Statistical significance was tested using the Wald test in cuffdiff and corrected for FDR using the Benjamini–Hochberg [19]. Data from Kummer et al. were reanalysed using GEO2R [23, 24]. The analysis utilised the Bioconductor/R *limma* package [25] with default settings, including log‐transformation of the data and FDR adjustment for multiple testing. Data from Calls et al. were processed in the original work. Statistical significance was tested using the default Seurat pipeline in *R* and corrected for FDR [7]. Statistical significance in the data set of Berta et al. [30] was tested by one‐way ANOVA with Bonferroni multiple comparison testing between each condition (^∗^ *p* < 0.05). All significance thresholds are indicated throughout the main text.

## 3. Results

### 3.1. *Cdkn1a* RNA Expression Exhibits a Recurrent Significant Increase in DRG Tissue Across Rodent Lesion‐Based Neuropathic Pain Models

It has recently been observed that inducing neuropathic pain in rodents leads to increased *Cdkn1a* (p21) and *Cdkn2a* (p16) RNA levels in DRG neurons, accompanied by an increased number of *Cdkn1a*‐ and *Cdkn2a*‐positive DRG neurons [8]. First, we sought to test whether this increase in *Cdkn1a* and *Cdkn2a* RNA is found across pain models and independent study settings. To this end, we made use of publicly available transcriptome data sets (Figure 1A).

**FIGURE 1:**
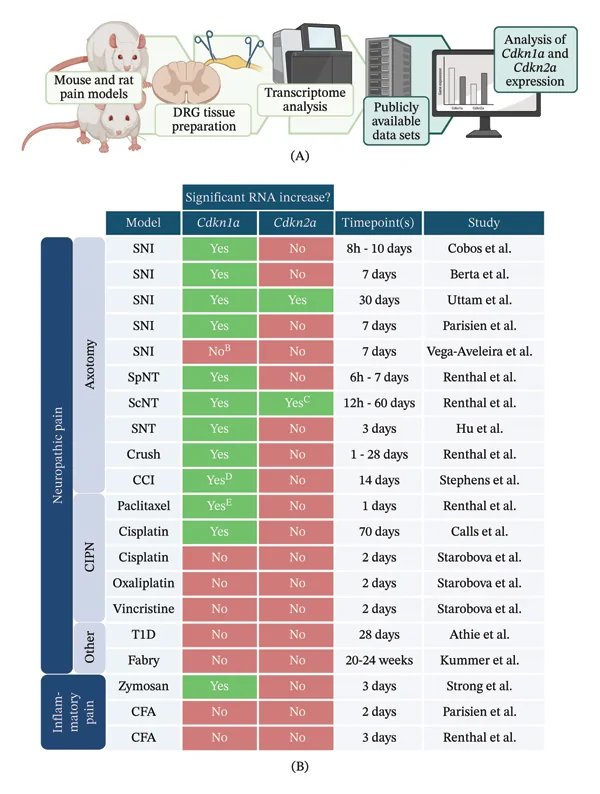
*Cdkn1a* (p21) RNA expression showing a recurrent significant increase in dorsal root ganglion tissue in rodent lesion‐based neuropathic pain models. (A) Schematic illustrating our study design with the performed workflow here in dark green, which builds on previously conducted transcriptome analyses of dorsal root ganglion (DRG) cell gene expression in mouse or rat pain models (light green). (B) Summarising table of significant *Cdkn1a* and *Cdkn2a* expression following our vote‐counting strategy. CIPN, chemotherapy‐induced peripheral neuropathy; SNI, spared nerve injury; SpNT, spinal nerve transection; ScNT, sciatic nerve transection and ligation; Crush, sciatic nerve crush; CCI, chronic constriction injury; T1D, streptozotocin‐induced Type 1 diabetes; Fabry, *α*‐galactosidase A deficiency as a Fabry disease model; CFA, complete Freund’s adjuvant. ^A^Not significant but *p*‐adj. = 0.051 and Log2FC = 2.88 with *n* = 3 samples. ^B^Only at 60 days post‐ScNT. ^C^Only in DRG tissue from female but not male rats. ^D^Only in *S* *s* *t* ^+^ pruriceptors but not in other DRG neuron subtypes.

We analysed 13 independent studies encompassing a total of 20 data sets of differential gene expression between rodent DRG‐derived cells derived from pain models and respective controls (Table 1). We followed a vote‐counting strategy based on the individual study thresholds, rather than a formal statistical integration, due to the heterogeneity of study designs including different sequencing platforms and inconsistent availability of gene expression data for genes above significance thresholds. Pain models included neuropathic pain models such as the SNI model or chemotherapy‐induced peripheral neuropathy (CIPN) and inflammatory pain models based on complete Freund’s adjuvant (CFA). *Cdkn1a* and *Cdkn2a* RNA levels were analysed by whole‐genome microarray, bulk‐RNA, single‐cell (sc) or single‐nucleus (sn) RNA sequencing methods. Timepoints of transcriptome analysis ranged from 6 h up to 70 days postinjury.

We found that *Cdkn1a* RNA shows a robust recurrent upregulation in DRG tissue in lesion‐based neuropathic pain models, reaching statistical significance in nine out of 10 data sets (Figure 1B). Significant *Cdkn1a* upregulation was observed at timepoints ranging from 6 h to 70 days postinjury (Figure 2). In contrast, *Cdkn2a* RNA is rarely significantly increased in DRG tissue. *Cdkn2a* RNA was only found to be significantly increased 30 days following SNI [12], and 60 days following sciatic nerve transection (ScNT) [16]. Interestingly, *Cdkn1a* RNA was less consistently increased in neuropathic pain models that do not rely on direct lesioning of nerve tissue such as CIPN, streptozotocin‐induced Type 1 diabetes and a genetic Fabry disease model, although all these models supposedly model neuropathic pain mechanisms (Figure 1B). In the context of inflammatory pain, *Cdkn1a* RNA was only significantly increased in one out of three inflammatory pain data sets (Figure 1B).

**FIGURE 2:**
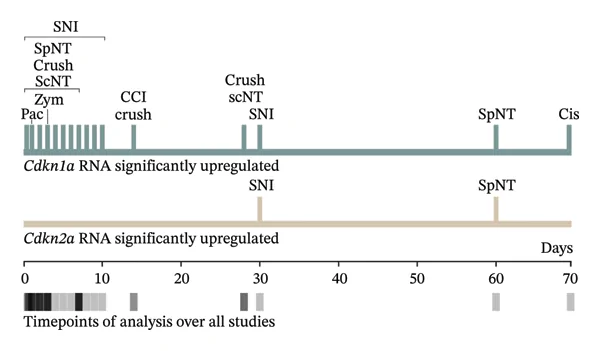
Significant *Cdkn1a* (p21) and *Cdkn2a* (p16) expression over time. Schematic showing the range of timepoints used for analysis across the 20 data sets spanning 6 h to 70 days postinjury. Grey bars depict distinct and accumulated analysis timepoints. Colorised vertical lines show timepoints of significant *Cdkn1a* or *Cdkn2a* expression together with the corresponding pain model. SNI, spared nerve injury; SpNT, spinal nerve transection; ScNT, sciatic nerve transection and ligation; Crush, sciatic nerve crush; CCI, chronic constriction injury; Pac, paclitaxel; Zym, zymosan; Cis, cisplatin.

### 3.2. *Cdkn1a* RNA Is Consistently Increased in Rodent DRG Tissue After Spared Nerve Injury

The most comprehensive work on peripheral sensory neuron senescence to date made use of the SNI model to study DRG neuron senescence and to test a potential pain‐resolving effect of senolytics [8]. To validate their findings, we now specifically investigated *Cdkn1a* and *Cdkn2a* RNA levels in independent studies that likewise used the SNI model.

In 2018, Cobos et al. performed whole‐genome microarray analysis with ipsilateral DRG tissue isolated from mice at multiple timepoints over the course of 10 days following SNI and naïve (control) mice [17]. The onset of cold and tactile allodynia within 5 days, which lasted for at least 15 days following SNI, was shown by acetone application and von Frey behavioural testing [17]. A total of 1704 microarray probes were found to be differentially regulated over the course of 10 days post‐SNI when compared to the expression in DRG tissue from naïve control mice (*p* < 0.01) [17]. Three probes were directed against distinct parts of *Cdkn1a* mRNA allowing to exclude nonspecific signals or technical errors, and all three probes showed significantly regulated RNA levels within the first 10 days following SNI (*F* = 9.009, *p* < 0.00001, *F* = 26.96, *p* < 0.00001, *F* = 19.64, *p* < 0.00001) (Figure 3A–C). The individual expression values indicated a trend towards early *Cdkn1a* upregulation within one day and a potential decline in *Cdkn1a* RNA from seven to 8 days post‐SNI. In contrast, *Cdkn2a* RNA was not significantly regulated within these 10 days [17].

**FIGURE 3:**
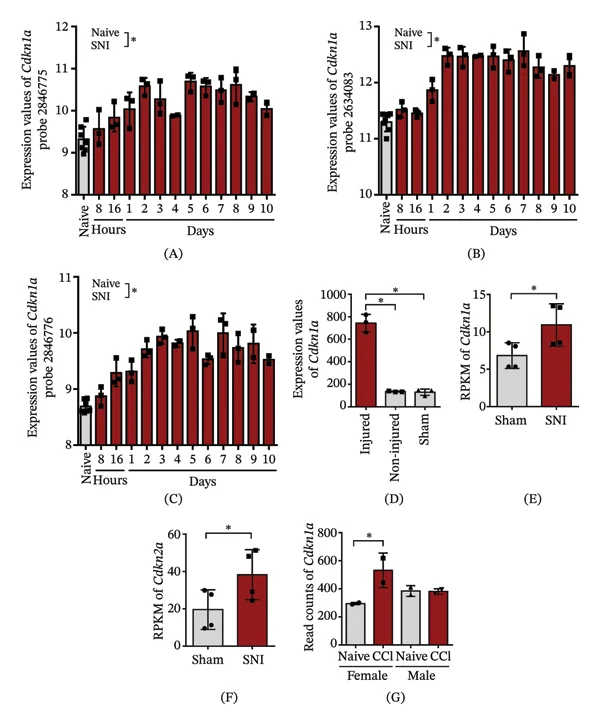
*Cdkn1a* (p21) RNA expression showing a recurrent increase in dorsal root ganglion tissue in rodent lesion‐based neuropathic pain models. (A–C) Expression values of the three *Cdkn1a*‐specific probes 2846775 (A), 2634083 (B) and 2846776 (C) over 10 days following spinal nerve injury (SNI) obtained from Cobos et al. [17]. Statistical significance was tested using moderated F‐statistic in the original study (^∗^ *p* < 0.01): (A) *F* = 9.009, *p* < 0.00001; (B) *F* = 26.96, *p* < 0.00001; (C) *F* = 19.64, *p* < 0.00001 [17]. (D) Expression values of *Cdkn1a* RNA in injured and noninjured DRG neurons and DRG neurons from sham‐operated animals 7 days postspinal nerve injury (SNI), obtained from Berta et al. [30]. Statistical significance was tested by one‐way ANOVA with Bonferroni multiple comparison testing between each condition (^∗^ *p* < 0.05). (E, F) Reads per kilobase of transcript per million mapped reads (RPKM) of *Cdkn1a* (B) and *Cdkn2a* (C) RNA in DRG tissue 30 days post‐SNI, obtained from Uttam et al. [12]. Statistical significance was tested in the original study and corrected for false discovery rates (FDR) using the Benjamini–Hochberg method (^∗^ *p* − adj.<0.05): (B) *p* − adj. = 0.0170; (C) *p* − adj. = 0.0030 [12]. (G) Read counts of *Cdkn1a* RNA in DRG tissue 14 days after chronic constriction injury (CCI), obtained from Stephens et al. [22]. Statistical significance was tested in the original study using the Wald test and corrected for false discovery rates (FDR) using the Benjamini–Hochberg (^∗^ *p* − adj.<0.05) [22]. (A–G) Data presented as individual data points and mean ± SD.

Parisien et al. also analysed gene expression changes following SNI [13]. The authors performed whole‐genome bulk‐RNA sequencing with lumbar DRG tissue from mice 7 days after bilateral SNI to model neuropathic pain [13]. This timepoint was selected because von Frey behavioural testing showed that induced hypersensitivity peaked at 7 days post‐SNI [13]. In DRG tissue at seven days post‐SNI, 1693 genes were significantly upregulated (*p* < 0.05, |*π*| ≥ 1.11) [13]. *Cdkn1a* but not *Cdkn2a* RNA was significantly increased at 7 days post‐SNI by 2.53‐fold (*p* < 0.00005, |*π*| = 20.735).

Berta et al. focused on gene expression changes 7 days post‐SNI as well [30]. They analysed RNA levels specifically in injured and noninjured rat DRG neurons [30]. For this, the authors combined microarray analysis with tracer‐guided laser capture microdissection [30]. *Cdkn1a* expression was 5.77‐fold higher in injured DRG neurons when compared to DRG neurons isolated from sham animals (Figure 3D). *Cdkn1a* expression was unaffected in noninjured DRG neurons (Figure 3D) [30].

A fourth study that investigated gene expression changes 7 days post‐SNI was performed by Vega‐Aveleira et al. [21]. Microarray analysis was conducted with ipsilateral and contralateral DRG tissue from rats 7 days after SNI or sham procedure [21]. The analysis was performed with two‐month‐ and ten‐day‐old rats, and resulted in 206, and three significantly regulated probes, respectively (*p* < 0.05). In this microarray data, neither *Cdkn1a* nor *Cdkn2a* expression was found to be significantly regulated following SNI [21]. However, reanalysing RNA levels in ipsilateral and contralateral DRG tissue showed a trend towards increased *Cdkn1a* RNA levels with a log2‐fold change of 2.88 and *p*‐adj. of 0.051 (*n* = 3 samples) in 2‐month‐old but not 10‐day‐old rats 7 days post‐SNI. Interestingly, a putative age‐dependent effect on *Cdkn1a* upregulation and induced senescence in DRG neurons has been previously also observed when comparing 10‐ to 16‐week‐old with 20‐ to 24‐month‐old mice following SNI [8].

In a fifth independent study using the SNI model, Uttam et al. performed whole‐genome bulk‐RNA sequencing with lumbar DRG tissue isolated from mice—this time 30 days—following bilateral SNI and mice that underwent sham surgery as a control [12]. Von Frey behavioural testing confirmed significantly reduced mechanical thresholds at 14, 21 and 30 days following SNI as an indication of chronic pain [12]. In their study, 144 mRNAs were found to be significantly increased 30 days following SNI (log2‐fold change > 0.5, *p* − *a* *d* *j*.<0.05) [12]. Among these, 144 mRNAs were *Cdkn1a* and *Cdkn2a* [12]. *Cdkn1a* RNA levels were significantly increased by 1.61‐fold in DRG tissue 30 days following SNI (*p* − *a* *d* *j*. = 0.0170), and *Cdkn2a* RNA levels were significantly increased by 1.72‐fold (*p* − *a* *d* *j*. = 0.0030) (Figure 3E, F).

Overall, significantly increased *Cdkn1a* RNA levels in mouse and rat DRG tissue following SNI are a consistent finding across independent study settings.

### 3.3. *Cdkn1a* RNA Is Not Consistently Increased Across Neuropathic Pain Models of Chemotherapy‐Induced Peripheral Neuropathy

In addition to surgical procedures, neuropathic pain can be modelled by CIPN using different drugs, including cisplatin, paclitaxel, vincristine and oxaliplatin. In 2021, Calls et al. performed scRNA sequencing analysis with DRG cells isolated from mice 10 weeks after weekly administration of cisplatin or saline solution [7]. Onset of mechanical allodynia after 3 weeks was shown by von Frey behavioural testing [7]. Differential gene expression analysis between DRG‐derived neurons after cell type clustering identified 122 significantly differentially expressed genes (*p* < 0.05), with *Cdkn1a* being the only gene with a *p* − adj.<0.01 after correcting for FDRs. *Cdkn1a* RNA was increased by 1.99‐fold in DRG neurons from cisplatin‐treated mice when compared to control mice (*p* − adj. = 0.0018). Increased *Cdkn1a* expression in DRG neurons was confirmed at the protein level (p21) by western blot analysis and immunocytochemistry [7]. Notably, the authors of this study analysed additional senescence‐associated markers such as *β*‐galactosidase activity and pH2AX and, to our knowledge, were the first to suggest that cisplatin may induce neuropathic pain through a DRG neuronal senescence‐like response [7]. *Cdkn2a* RNA levels were not significantly altered in the cisplatin model.

Starobova et al. performed whole‐genome bulk‐RNA sequencing analysis with DRG tissue from mice 2 days after vincristine, oxaliplatin or cisplatin injection, and control mice as three different models for CIPN [18]. Vincristine led to 368, oxaliplatin to 295 and cisplatin to 256 significantly differentially expressed genes (*p* − adj.<0.05) [18]. *Cdkn1a* and *Cdkn2a* were not significantly differentially expressed in this study, which performed analyses at 2 days following compound injection. Given that Calls et al. observed significantly increased *Cdkn1a* expression 10 weeks following continuous cisplatin treatment [7], the timepoint of analysis may play an important role when analysing CIPN‐induced *Cdkn1a* expression.

### 3.4. *Cdkn1a* RNA Is Significantly Increased Only in DRG Tissue From Female but Not Male Rats 14 Days After Chronic Constriction Injury (CCI)

Previously, the female sex has been associated with a greater likelihood of senescence onset, although specific evidence is lacking (reviewed in [31]). Sex‐specific differences in DRG neuron senescence have not been described.

Hence, we searched for prior sex‐specific analyses of DRG neuron gene expression in the context of neuropathic pain to analyse *Cdkn1a* and *Cdkn2a* RNA expression. In 2019, Stephens et al. performed sex‐specific whole‐genome bulk‐RNA sequencing analysis with DRG tissue from male and female rats 14 days after CCI and respective male and female untreated (naïve) controls [22]. Mechanical hypersensitivity at Day 14 post‐CCI was confirmed by von Frey behavioural testing [22].

The authors observed that 1185 genes were significantly upregulated in males but not in females, and that 146 genes were significantly upregulated in females but not in males upon CCI (log2FC > 0.5, *p* − adj.<0.05). Among these, 146 female‐specific upregulated genes included *Cdkn1a*. *Cdkn1a* RNA levels were significantly increased by 2.06‐fold (*p* − adj.<0.0001) in female rats 14 days post‐CCI when compared to female naïve controls, but not significantly increased in male rats when compared to male naïve controls (Figure 3G). This bulk‐RNA sequencing data set was validated by *Cdkn1a*‐specific RT‐qPCR with a separate set of DRG tissue preparations. Interestingly, there was no significant sex‐specific difference in *Cdkn1a* RNA levels found in DRG tissue derived from naïve rats [22]. Hence, these data by Stephens et al. may indicate that *Cdkn1a* expression is differentially regulated in DRG neurons in male and female rats.

Since this is a single study showing increased *Cdkn1a* RNA levels in DRG cells derived from female but not from male rats, general conclusions about a potential sex‐specific *Cdkn1a* regulation in response to neuronal injury cannot be drawn. However, this observation warrants further investigation.

### 3.5. *Cdkn1a* RNA Is Increased in All Mouse DRG Neuronal Subtypes and to a Lesser Extent in Non‐Neuronal Cells Across Neuropathic Pain Conditions

So far, we have mostly analysed data from microarray or bulk‐RNA sequencing studies that do not allow differentiation between *Cdkn1a* or *Cdkn2a* RNA in DRG‐resident neuronal subtypes or associated non‐neuronal cells. To investigate whether *Cdkn1a* or *Cdkn2a* expression may predominantly occur in certain DRG neuron subtypes or additionally in non‐neuronal cells, we searched for studies that performed single‐cell analysis with neuronal subtype‐specific downstream analysis.

In 2016, Hu et al. performed an initial whole‐genome bulk‐RNA sequencing analysis with pooled neurons from single‐cell preparation derived from ipsilateral and contralateral DRG tissue isolated from mice 3 days after ScNT as a control for their subsequent scRNA analysis [15]. This initial bulk‐RNA sequencing experiment showed that 1179 genes were differentially expressed at 3 days post‐SNT in the ipsilateral DRG tissue when compared to the contralateral (control) DRG tissue (fold change > 2, *p* − adj.<0.05). Among these, 624 genes were upregulated, including *Cdkn1a*. *Cdkn1a* RNA was significantly increased by 18.89‐fold in this bulk‐RNA analysis of pooled DRG neurons, already showing that *Cdkn1a* expression is indeed induced in DRG neurons (*p* − adj. = 0.0041).

Afterwards, the authors performed scRNA sequencing analysis based on the same experimental setting of isolating DRG tissue 3 days post‐SNT. This way, differential gene expression was analysed individually in peptidergic nociceptors (222 upregulated, 181 downregulated), in nonpeptidergic (NP) nociceptors (1188 upregulated, 1067 downregulated), and in large myelinated sensory neurons (51 upregulated, 32 downregulated). *Cdkn1a* RNA was significantly increased in all three major sensory neuron populations at 3 days post‐SNT: peptidergic nociceptors = 3.86 log2‐fold change, *p* − adj.<0.00005; NP nociceptors = 4.34 log2‐fold change, *p* − adj.<0.00005; large myelinated sensory neurons = 5.38 log2‐fold change, *p* − adj.<0.00005.

In the most comprehensive scRNA analysis in our context, Renthal et al. performed differential gene expression analysis with nine DRG neuronal subtypes and with DRG‐resident non‐neuronal cells. The authors performed snRNA sequencing analysis with DRG tissue across several pain models to map gene expression profiles in response to lesion‐based nerve injury and CIPN [16]. For this, lumbar DRG tissue was isolated from adult mice following spinal nerve transection (SpNT), sciatic nerve crush (Crush), ScNT and ligation, and paclitaxel at various timepoints. Differential gene expression (*p* − adj.<0.01 and log2fc > |1|) was captured between DRG cells from naïve (untreated) mice and their respective pain models within *F* *a* *m*19*a*4^+^/*T* *h* ^+^ C‐fibre low‐threshold mechanoreceptor (cLTMR1) neurons, putative *F* *a* *m*19*a*4^+^/*T* *h* ^low^ cLTMR2 neurons, *M* *r* *g* *p* *r* *d* ^+^ NP nociceptors, *P* *v* *a* *l* *b* ^+^ proprioceptors (NF1, NF2), *C* *a* *d* *p* *s*2^+^ Ad‐LTMR (NF3) neurons, *T* *a* *c*1^+^/*G* *p* *x*3^+^ peptidergic nociceptors (PEP1), *T* *a* *c*1^+^/*H* *p* *c* *a* ^+^ peptidergic nociceptors (PEP2) and *S* *s* *t* ^+^ pruriceptors (SST) [16]. Furthermore, differential gene expression within DRG‐resident myelinating Schwann cells, nonmyelinating (Remark) Schwann cells, macrophages, endothelial cells and fibroblasts was analysed [16].

*Cdkn1a* expression was significantly increased in all analysed DRG neuron subtypes across the three lesion‐based neuropathic pain models, SpNT (Figure 4), Crush (Figure 5) and ScNT (Figure 6). This increase occurred as early as 6 h after surgical intervention in some DRG neuron subtypes and remained significantly increased for as long as 60 days after ScNT in NF2 neurons. The data clearly show that *Cdkn1a* RNA levels peak after three to seven days in the Crush and scNT model. Through the different timepoints of analysis postinjury in this study, it is difficult to conclude whether the three nerve injury models differ with regard to induced *Cdkn1a* levels. *Cdkn1a* expression was significantly increased also in the non‐neuronal cell types, although to a lesser extent and not as consistently as in neurons. At 24 days post‐paclitaxel application as a model for CIPN, *Cdkn1a* expression was only significantly increased in *S* *s* *t* ^+^ pruriceptors (SST) in this work (log2fc = 1.56, *p* − adj.<0.00001).

**FIGURE 4:**
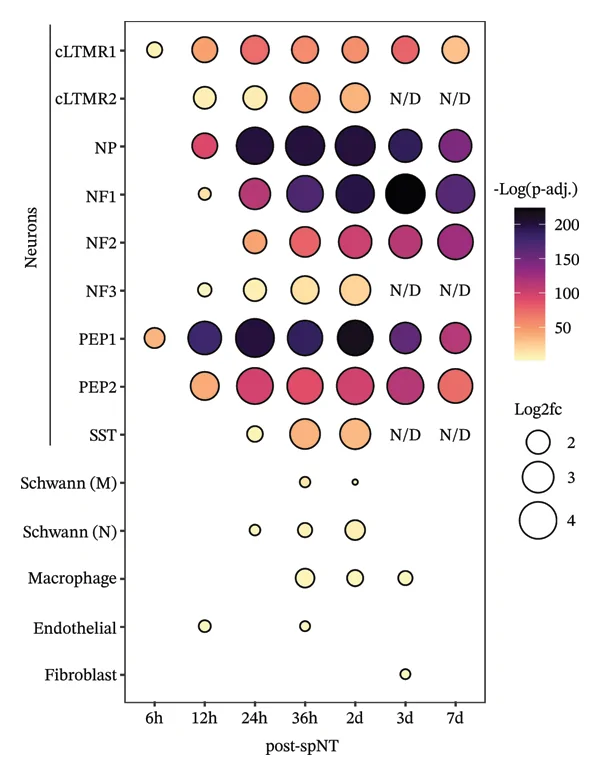
*Cdkn1a* (p21) RNA is increased in all dorsal root ganglion neuronal subtypes following spinal nerve transection in Renthal et al. Bubble heatmap of significantly increased *Cdkn1a* RNA expression as log2‐fold change and −log10(*p* − adj.) in mouse dorsal root ganglion (DRG) cells following spinal nerve transection (spNT) when compared to expression in DRG cells from naïve mice, as obtained from Renthal et al. [16]. Differential *Cdkn1a* gene expression is only shown when *p* − adj.<0.01 and log2fc > |1| when compared to expression in cells from naïve control mice. Statistical significance was tested in the original manuscript using the QL F‐test in edgeR [16]. N/D = no data for these timepoints because too few cells were available to perform cell type‐specific differential gene expression analysis at these individual timepoints.

**FIGURE 5:**
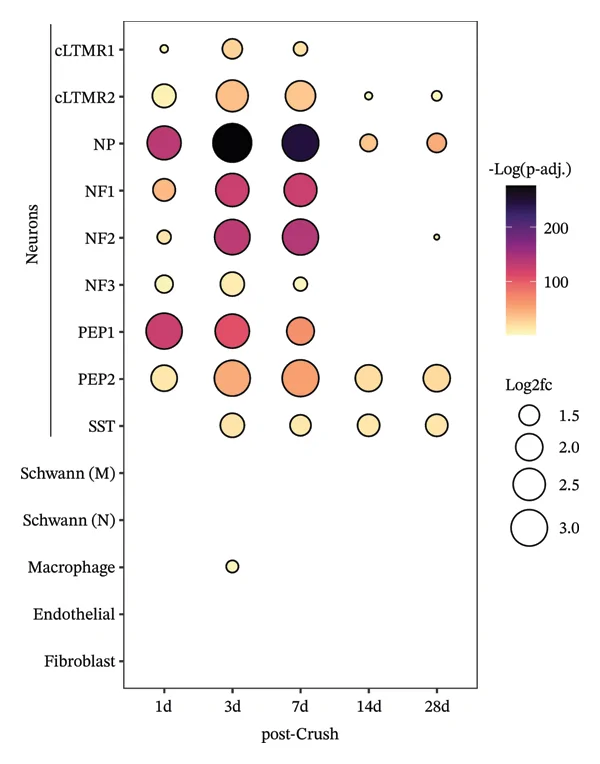
*Cdkn1a* (p21) RNA is increased in all dorsal root ganglion neuronal subtypes following nerve crush in Renthal et al. (A–C) Bubble heatmap of significantly increased *Cdkn1a* RNA expression as log2‐fold change and −log10(*p* − adj.) in mouse dorsal root ganglion (DRG) cells following sciatic nerve crush (Crush) when compared to expression in DRG cells from naïve mice, as obtained from Renthal et al. [16]. Differential *Cdkn1a* gene expression is only shown when *p* − adj.<0.01 and log2fc > |1| when compared to expression in cells from naïve control mice. Statistical significance was tested in the original manuscript using the QL F‐test in edgeR [16].

**FIGURE 6:**
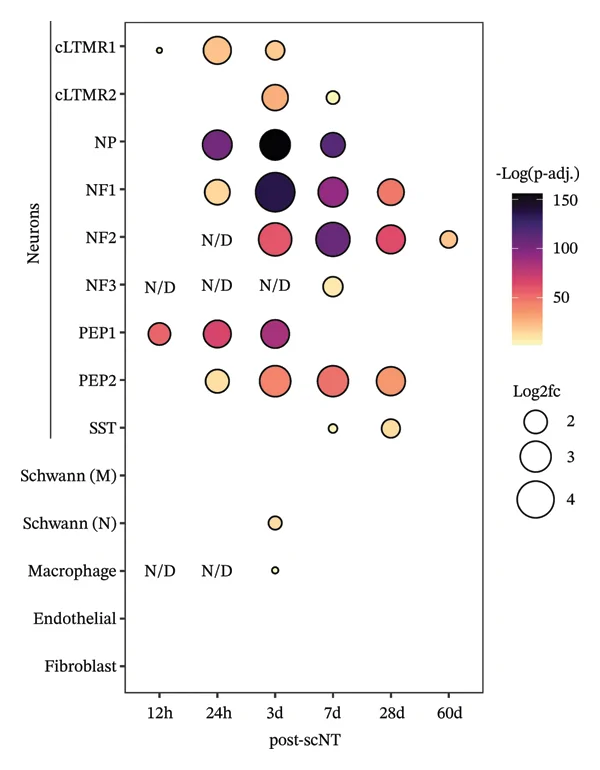
*Cdkn1a* (p21) RNA is increased in all dorsal root ganglion neuronal subtypes following sciatic nerve transection in Renthal et al. (A–C) Bubble heatmap of significantly increased *Cdkn1a* RNA expression as log2‐fold change and −log10(*p* − adj.) in mouse dorsal root ganglion (DRG) cells following sciatic nerve transection and ligation (ScNT) when compared to expression in DRG cells from naïve mice, as obtained from Renthal et al. [16]. Differential *Cdkn1a* gene expression is only shown when *p* − adj.<0.01 and log2fc > |1| when compared to expression in cells from naïve control mice. Statistical significance was tested in the original manuscript using the QL F‐test in edgeR [16]. N/D = not data for these timepoints because too few cells were available to perform cell type‐specific differential gene expression analysis at these individual timepoints.

In the whole study setting, *Cdkn2a* expression was only significantly increased in NF2 neurons at 60 days post‐ScNT (log2fc = 1.30, *p* − adj.<0.00001).

Due to the varying timepoints, pain models, interventions, sequencing pipelines and downstream analyses that underly the available data sets, it is not possible to calculate any mean fold changes of *Cdkn1a* RNA levels by formal analysis. Moreover, cell type composition effects mask actual peripheral sensory neuron *Cdkn1a* expression levels in bulk‐RNA experiments. Still, we concluded that *Cdkn1a* RNA levels may peak around Day 3 to Day 7 following nerve injury, and throughout this work, we have analysed three independent studies encompassing eight different differential gene expression data sets on rodent DRG neuron‐specific transcriptomes at either Day 3 or Day 7 after lesion‐based nerve injury through surgical intervention (SNI, SNT, SpNT, ScNT and Crush) [15, 16, 30]. Summarising these data sets showed that *Cdkn1a* expression in peripheral sensory neurons may increase by between 4.53‐ and 18.89‐fold following axonal lesion (Table 2).

**TABLE 2:** Differential *Cdkn1a* RNA expression in peripheral sensory neurons at three and 7 days postlesion.

Study	Analysed material	Days postlesion	Pain model	Fold change^A^
Berta et al.	Injured neurons	7	SNI	5.77

Hu et al.	Pooled neurons	3	SNT	18.89

Renthal et al.	Neuronal subtypes	3	SpNT	10.06
	Neuronal subtypes	7	SpNT	9.71
	Neuronal subtypes	3	Crush	5.31
	Neuronal subtypes	7	Crush	4.53
	Neuronal subtypes	3	ScNT	7.31
	Neuronal subtypes	7	ScNT	4.53

^A^The fold changes of *Cdkn1a* expression changes per neuronal subtype in the Renthal et al. study were averaged to obtain the overall fold change for all DRG neurons.

Overall, these data sets clearly show that DRG neurons express increased *Cdkn1a* RNA levels in response to axonal lesions, as indicated by the previously described bulk‐RNA sequencing and microarray‐based studies. Furthermore, it suggests that *Cdkn1a* expression is increased in all identified DRG neuron subtypes. At last, this study corroborates that *Cdkn2a* is not effectively upregulated following peripheral nerve injury in DRG neurons.

### 3.6. *Cdkn1a* and *Cdkn2a* RNA Is Not Significantly Increased Across Inflammatory Pain Models

A prior study suggested that modelling inflammatory pain with CFA leads to senescence of DRG neurons as early as 2 days following injection [9]. To test whether inducing inflammatory pain is accompanied by increased *Cdkn1a* or *Cdkn2a* RNA in different study settings, we searched for transcriptome studies that applied inflammatory pain models. Parisien et al. performed whole‐genome bulk‐RNA sequencing with lumbar DRG tissue from mice 3 days after CFA application and naive mice as control [13]. This timepoint was selected because von Frey behavioural testing showed that induced hypersensitivity peaked at 3 days post‐CFA [13]. At this timepoint, 839 genes were significantly upregulated in DRG tissue. However, neither *Cdkn1a* nor *Cdkn2a* RNA levels were significantly increased at 3 days post‐CFA in this study [13].

In the previously described study that performed snRNA analysis to investigate gene expression changes in nine DRG neuronal subtypes and non‐neuronal cells across four different neuropathic pain models (SpNT, ScNT, Crush, Paclitaxel), the authors additionally analysed gene expression 2 days following CFA injection as a model for inflammatory pain. Although *Cdkn1a* RNA was significantly increased in DRG cells in their neuropathic pain models, *Cdkna1a* expression was not significantly increased 2 days post‐CFA application [16].

Another model of inflammatory pain is injecting the immune activator zymosan. Strong et al. performed microarray analysis with DRG tissue from rats 3 days after injection with zymosan in incomplete Freund’s adjuvant beneath the intervertebral foramen onto the L5 DRG to model inflammatory pain and from sham‐treated rats as control [14]. Mechanical hypersensitivity at 3 days postinjection in zymosan‐injected animals, and lack of hypersensitivity in sham animals, was confirmed by von Frey behavioural testing [14]. In DRG tissue at 3 days postzymosan injection, 1625 out of 6831 captured genes showed significantly differential RNA levels (*p* − adj.<0.05), including *Cdkn1a* [14]. *Cdkn1a* RNA was increased by 1.37‐fold in DRG tissue after zymosan injection when compared to DRG tissue from sham‐treated rats (*p* − adj. = 0.016). *Cdkn2a* RNA levels were not significantly increased at 3 days postinjection.

Thus, in contrast to changes in *Cdkn1a* RNA in neuropathic pain models, results are less consistent between studies investigating inflammatory pain. It remains unclear whether inducing inflammatory pain in rodents consistently leads to increased *Cdkn1a* in DRG tissue and whether it may at all lead to increased *Cdkn2a* RNA levels.

### 3.7. *Cdkn1a* and *Cdkn2a* RNA Is Not Significantly Increased in All Studies and Pain Models

We found two additional data sets in which neither *Cdkn1a* nor *Cdkn2a* RNA was significantly increased in rodent DRG tissue upon induction of peripheral‐driven pain. In the context of diabetic neuropathy, Athie et al. performed whole‐genome bulk‐RNA sequencing analysis with DRG tissue from rats 4 weeks following induction of Type 1 diabetes with streptozotocin and control rats [19]. Induction of Type 1 diabetes resulted in mechanical hypersensitivity from Day 14 on as determined by von Frey behavioural testing and led to 56 significantly expressed genes in DRG cells after 4 weeks (*p* − adj.<0.05). *Cdkn1a* and *Cdkn2a* were not significantly differentially expressed [19]. Kummer et al. performed microarray analysis with DRG tissue isolated from *α*‐galactosidase A–deficient and wild‐type mice as a model for Fabry disease–related neuropathic pain [20]. In total, 812 genes were significantly differentially expressed between DRG tissue from *α*‐galactosidase A–deficient and wild‐type mice (|Fold change| ≥ 1.2, *p* − adj.<0.01) [20]. *Cdkn1a* and *Cdkn2a* were not significantly differentially expressed in DRG tissue in this Fabry disease model.

## 4. Discussion

Peripheral sensory neuron senescence has recently been associated with increased nociceptive signalling and suggested as a target for novel pain‐resolving strategies. By analysing publicly available transcriptomic data sets, we found that the widely used marker for cellular senescence *Cdkn1a* is consistently upregulated in DRG tissue—specifically in DRG neurons—in lesion‐based neuropathic but less consistently in other pain models (Figure 7).

**FIGURE 7:**
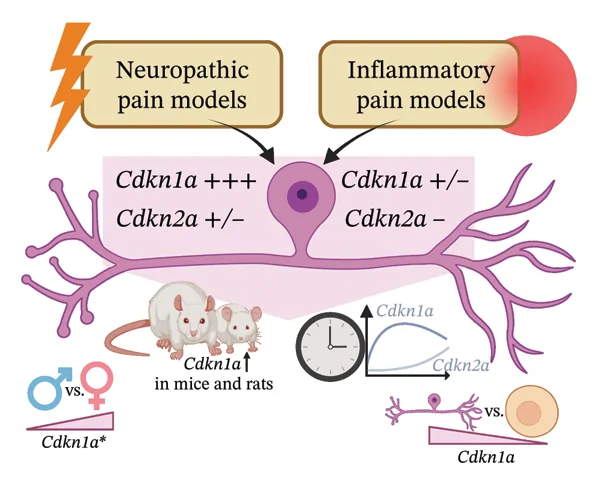
*Cdkn1a* RNA showing a robust recurrent significant increase in lesion‐based neuropathic pain models. Concluding schematic summarising key aspects indicated by our analysis of publicly available transcriptomic data sets: *Cdkn1a* RNA may be increased to a higher extent in DRG neurons from females upon nerve injury (^∗^This conclusion is based on a single data set). *Cdkn1a* RNA is increased in DRG cells from mice and rats in pain conditions. *Cdkn1a* RNA is increased within hours to days following nerve injury, while *Cdkn2a* RNA is—if at all—increased only at later timepoints. *Cdkn1a* RNA is increased primarily in DRG neurons and to a lesser extent and less consistently in non‐neuronal cells in pain conditions.

We observed that *Cdkn1a* expression is upregulated in DRG neurons in less than a week in lesion‐based neuropathic pain models. This is in accordance with other model systems of induced senescence such as drug‐induced senescence in cell culture, in which CDKN1A (p21) upregulation can be detected within few hours and steadily increases over the first days [32].

Interestingly, CDKN1A (p21) expression induced in cancer cells by chemotherapy was found to have the capacity to induce either senescence or proliferation [11]. An early increase in p21 did not automatically lead to cellular senescence [11]. Thus, *Cdkn1a* expression at these early timepoints alone may not guarantee peripheral sensory neuron senescence.

Based on our analysis of data by Renthal et al. [16], we observed that *Cdkn1a* RNA expression levels peak around 3 to 7 days after induction of neuropathic pain. Afterwards, RNA expression starts to decline and may return to baseline in some DRG neuronal subtypes over 28 days, but may remain significantly elevated as long as 60 days postinjury. This is similar to observations for other cell types such as fibroblasts [33]. *Cdkn1a* expression appears to increase during the early phase but subsequently declines once cellular senescence is established. At this stage, senescence‐maintaining mechanisms predominate, and SASP develops [2, 33].

The same scRNA‐based analysis also clearly showed that all DRG‐resident neurons express increased *Cdkn1a* RNA levels in lesion‐based pain. Non‐neuronal cell types showed increased *Cdkn1a* levels as well, but this increase was not as profound as in neurons. Thus, it can be assumed that the increase in neuronal *Cdkn1a* expression may be masked in bulk RNA‐seq and microarray analysis. Moreover, these observations show that the increased *Cdkn1a* expression levels observed in the bulk RNA‐seq and microarray experiments are not due to cell type composition effects, e.g., through infiltrating immune cells, but are a neuronal response mechanism in the DRG.

Our analysis showed that *Cdkn2a* expression was significantly increased only 30 days post‐SNI or 60 days post‐ScNT, but not in the studies that performed differential gene expression analysis 7 days post‐SNI. Hence, increased *Cdkn2a* expression is—if at all—observed at later timepoints in DRG neurons of neuropathic pain models. In a previous study, Donovan et al. likewise observed significantly increased numbers of *Cdkn2a*‐positive DRG neurons only at 3 weeks, but not at 7 days post‐SNI, when analysing 11‐ to 16‐week‐old mice [8]. In contrast, the numbers of *Cdkn1a*‐positive neurons were significantly increased already at 7 days post‐SNI in their study [8]. The data sets analysed here seem to corroborate such a delayed *Cdkn2a* upregulation that occurs after *Cdkn1a* upregulation following SNI in DRG cells, which is a common observation in the context of injury‐induced senescence and may be due to the different functions of the two gene products [33–35]. On the other hand, there is also evidence that distinct cell types and subpopulations either predominantly express high *Cdkn1a* or high *Cdkn2a* levels [35]. Indeed, CDKN2A expression has been previously pointed out as not as reliable as CDKN1A to monitor senescence of central nervous system neurons [2].

We then found preliminary indications that *Cdkn1a* upregulation in DRG tissue in response to nerve injury may be higher in females than in males. This was based on a single study analysing differential gene expression in male and female rats following CCI, and therefore, this is not definitive evidence. However, sex‐specific gene expression has been reported in rodent DRG neurons, and in females, DRG neuron gene expression is influenced by hormonal status [36, 37]. Hence, it is possible that *Cdkn1a* may be differentially regulated sensory neurons in male and female rats. Accordingly, a previous study showed that etoposide‐induced DNA damage led to higher levels of *Cdkn1a* mRNA in astrocytes from female than from male mice [38]. Additional evidence for increased *Cdkn1a* upregulation in female cells is lacking, because most transcriptomic studies include either male or female animals, but not both. Therefore, we argue that our observation calls for more research on potential sex‐specific peripheral sensory neuronal senescence. Moreover, future studies should include female and male animals, which is often not the case (Table 1), and, if possible, investigate possible sex differences.

Besides sex as a possible variable influencing *Cdkn1a* or *Cdkn2a* expression, it may be possible that age influences neuronal responses. We found that *Cdkn1a* RNA levels showed a trend towards upregulation in DRG tissue derived from 2‐month‐old but not 10‐day‐old rats 7 days post‐SNI. This is in line with a putative age‐dependent effect on *Cdkn1a* upregulation and induced senescence in DRG neurons that has been observed when comparing 10‐ to 16‐week‐old with 20‐ to 24‐month‐old mice following SNI [8]. Together, these observations may indicate that age is a potential factor in *Cdkn1a* regulation, and, therefore, may present age as a possible confounder that needs to be taken into account in future study designs addressing *Cdkn1a* regulation or peripheral neuron senescence.

Given the increase in *Cdkn1a* RNA in DRG neurons across lesion‐based neuropathic pain models found in this study, the previously indicated role for Cdkn1a as a necessary signal transducer to induce neuronal senescence [4], and the association of senolytic therapy with reduced nocifensive behaviour in animal pain models [8, 9], it would be interesting to test conditional Cdkn1a (p21) knockout mice in such pain models and whether pain chronification is diminished following induction of neuropathic pain. On the other hand, p21‐overexpressing (*C* *d* *k* *n*1*a* ^super^) mice exist, and it would be of great interest to test whether these animals suffer from worse pain phenotypes than wild‐type mice based on potentially increased rates of peripheral sensory neuron senescence [39]. Such experimental studies would further corroborate the putative role of Cdkn1a in peripheral‐driven pain chronification.

We observed a recurrent significant increase in *Cdkn1a* expression across lesion‐based, but not in other neuropathic pain models such as CPIN, diabetic neuropathy and Fabry disease. Cdkn1a as a cyclin‐dependent kinase inhibitor is involved in different neuronal cell cycle–related pathways [3]. Hence, it is plausible that peripheral sensory neuron injury through axotomy or axonal lesions is a stronger inducer of *Cdkn1a* expression than non–lesion‐based models. As outlined before, whether this induced *Cdkn1a* expression is causally linked to peripheral sensory neuron senescence or whether it is involved in neuronal regeneration functionally unrelated to senescence remains to be determined.

An inherent limitation of our cross‐study comparison is the substantial heterogeneity among the analysed data sets, with respect to included species, sexes, ages, timepoints of analysis and pain models. Even within single studies like the comprehensive snRNA sequencing work by Renthal et al. [16], timepoints for differential gene expression analysis following the different interventions are not the same. Furthermore, variations in sequencing platform, ranging from microarrays to next‐generation sequencing with single‐cell resolution, introduce technical variables. However, this heterogeneity highlights the robustness of our findings revealing increased *Cdkn1a* levels in lesion‐based pain models across independent data sets. Notably, there are fewer transcriptome studies that have analysed DRG cell gene expression responses in the context of inflammatory pain. This is especially true for later timepoints that may model late‐stage/chronic pain, for which data points in general are lacking, but which are completely absent after CFA or zymosan application. Thus, our picture of *Cdkn1a* and *Cdkn2a* expression in the context of peripheral pain is still incomplete. In addition, due to the heterogeneity of data sets, we relied on the statistical significance calls validated by the original authors (vote‐counting strategy) and did not perform a formal effect size analysis. We believe that this constitutes the most scientifically rigorous approach to map differential *Cdkn1a* and *Cdkn2a* expression across independent, highly diverse data sets. *Cdkn1a* and *Cdkn2a* expression levels are two well‐established and widely used markers for cellular senescence. Yet, their expression levels alone are not enough to conclude and define induction of neuronal senescence [3, 10]. We were careful with our interpretations and think that our study provides a starting point, but that future studies generating transcriptome data sets must analyse additional senescence‐ and SASP‐related factors such as TNF, IL‐6, H2AX, IL‐1*β*, SERPINE1, TP53 and CCL2. In addition, transcriptomic studies provide an incomplete picture of biological pathways because RNA levels do not necessarily correlate with protein level and thus with functionality [40]. Similarly, significantly increased *Cdkn1a* levels were validated by RT‐qPCR only in a single study [22], and additional senescence‐associated markers such as *β*‐galactosidase activity and pH2AX were analysed in another single study [7]. Future mechanistic studies addressing peripheral sensory neuron senescence must follow these examples and validate findings at the protein level and via functional assays. We focused on rodent pain models, because these studies are more consistent and better comparable than the few existing studies with human tissue with regard to our purpose of comparing *Cdkn1a* and *Cdkn2a* expression across different study settings. Thus, to assess the translational relevance of the here observed peripheral sensory neuron injury‐associated *Cdkn1a* expression, human samples need to be analysed in future work. Lastly, our conclusions may be influenced by publication bias, because negative transcriptomic data sets may not be published or reported. Consequently, while our findings offer valuable benchmarks to guide future study designs, they do not provide definitive evidence regarding the absolute presence or absence of differential *Cdkn1a* or *Cdkn2a* expression.

Overall, we conclude that an increase in *Cdkn1a* expression in DRG neurons is consistently found across rodent lesion‐based neuropathic pain models. It seems that increased *Cdkn1a* expression is not as often found in pain models beyond lesion‐based nerve injury. The timepoint of analysis, the age and the sex of the employed model animals may influence *Cdkn1a* gene expression.

**Nomenclature**
CCI: Chronic constriction injury
CFA: Complete Freund’s adjuvant
CIPN: Chemotherapy‐induced peripheral neuropathy
DRG: Dorsal root ganglion
FDR: False discovery rate
GEO: Gene Expression Omnibus
LCM: Laser capture microdissection
SASP: Senescence‐associated secretory phenotype
ScNT: Sciatic nerve transection
SNI: Spared nerve injury
SpNT: Spinal nerve transection

## Author Contributions

This study was designed by Philipp N. Ostermann and Tim Hucho. The data were analysed by Philipp N. Ostermann, Rebecca Dinnendahl and Jörg Isensee, and the results were critically examined by all authors. Philipp N. Ostermann had a primary role in preparing the manuscript, which was edited by Rebecca Dinnendahl, Jörg Isensee and Tim Hucho.

## Funding

This work was supported by the Deutsche Forschungsgemeinschaft, 516750869; Universität zu Köln.

## Disclosure

Versions of this manuscript have been placed on a preprint server: https://doi.org/10.64898/2026.01.20.700573 (Ostermann et al., 2026). All authors have approved the final version of the manuscript and agree to be accountable for all aspects of the work.

## Conflicts of Interest

The authors declare no conflicts of interest.

## Supporting Information

Additional supporting information can be found online in the Supporting Information section.

## Supporting information


**Supporting Information** Supporting figure 1: Flow diagram for systemic search of relevant gene expression data sets.

## Data Availability

The data that support the findings of this study are available from the corresponding author upon reasonable request.
