# Supplementary material for: Senescence‐Associated Cdkn1a (p21) Is Upregulated in Rodent Dorsal Root Ganglion Neurons Across Lesion‐Based Neuropathic Pain Models
Source: Pain Res Manag. 2026 Oct 25;2026:6836731. doi: 10.1155/prm/6836731 (PMC13695873; doi:10.1155/prm/6836731)
Supplement: Supplementary file 1 [file PRM-2026-6836731-s001.docx]

**Supplementary Fig 1. Flow diagram illustrating the applied systemic data set selection methodology**. DRG = Dorsal root ganglion; scRNA = single-cell RNA; snRNA = single-nucleus RNA.
